# Increased CDCA2 Level Was Related to Poor Prognosis in Hepatocellular Carcinoma and Associated With Up-Regulation of Immune Checkpoints

**DOI:** 10.3389/fmed.2021.773724

**Published:** 2022-03-07

**Authors:** Mengying Tang, Mingchu Liao, Xiaohong Ai, Guicheng He

**Affiliations:** ^1^Department of Infectious Disease, The First Affiliated Hospital, Hengyang Medical School, University of South China, Hengyang, China; ^2^Department of Medical Oncology, The First Affiliated Hospital, Hengyang Medical School, University of South China, Hengyang, China; ^3^Department of Radiation Oncology, The First Affiliated Hospital, Hengyang Medical School, University of South China, Hengyang, China

**Keywords:** liver cancer, hepatocellular carcinoma, CDCA2, survival, prognosis

## Abstract

**Background:**

Cell division cycle-associated protein 2 (CDCA2) is a member of cell cycle-related proteins. CDCA2 plays a role in the regulation of protein phosphatase 1(PP1) γ-dependent DNA damage response (DDR) and H3 phosphorylation. CDCA2 promotes the tumorigenesis and development of several types of cancers by promoting the proliferation of tumor cells. However, the relationship between CDCA2 expression and the clinicopathological characteristics of hepatocellular carcinoma (HCC) is unknown.

**Methods:**

Gene expression information and clinical data were downloaded from The Cancer Genome Atlas (TCGA) database. The expression of CDCA2 and its correlation to clinical characteristics in HCC were analyzed. The expression level of CDCA2 was validated in HCC cell lines. The relationship between CDCA2 expression and the survival of patients with HCC was analyzed by using Kaplan–Meier method. The prognostic value of CDCA2 in HCC was estimated by Cox regression analysis. The expression difference of CDCA2 between HCC and normal tissues and its correlation to survival were verified in independent datasets. Gene set enrichment analysis (GSEA) was used to screen the CDCA2-related signaling pathways.

**Results:**

Cell division cycle-associated protein 2 expression was upregulated in HCC tissues (*p* < 0.001) and increased CDCA2 was correlated to increased T stage, pathologic stage, histologic grade, and alpha-fetoprotein (AFP) level (*p* < 0.001). In addition, CDCA2 was overexpressed in HCC cell lines HepG2 and LM3. High CDCA2 expression level was associated with poor overall survival [hazard ratio (*HR*) = 1.69; 95% *CI*, 1.20–1.40, *p* = 0.003], disease specific survival (*HR* = 1.73; 95% *CI*, 1.11–2.71, *p* = 0.016), and progress free interval (*HR* = 1.74; 95% *CI*, 1.30–2.34, *p* < 0.001). Overexpression of CDCA2 and its correlation to poor survival in HCC were verified in Gene Expression Omnibus (GEO) datasets and Kaplan–Meier plotter database. Increased CDCA2 expression was associated with upregulation of PD-L1 (Spearman's coefficient = 0.207, *p* < 0.001), PD-L2 (Spearman coefficient's = 0.118, *p* < 0.05), and CTLA4 (Spearman's coefficient = 0.355, *p* < 0.001). GSEA showed that homologous recombination pathway, insulin signaling pathway, mitogen-activated protein kinase (MAPK) pathway, mismatch repair pathway, mechanistic target of rapamycin (mTOR) pathway, Notch pathway, T cell receptor pathway, toll like receptor pathway, and WNT pathway were enriched in CDCA2 high expression phenotype.

**Conclusion:**

Cell division cycle-associated protein 2 may serve as an independent biomarker for poor prognosis in HCC and increased CDCA2 expression was associated with upregulation of immune checkpoints.

## Introduction

Hepatocellular carcinoma (HCC) is one of the most common malignant tumors in the world, with a high cancer-related mortality. Each year, there are about 840,000 new cases of HCC and about 780,000 HCC related-deaths worldwide. The prognosis of HCC is poor, with a survival interval of 6–20 months without treatment (1, 2). For patients with resectable disease, surgical resection is the recommended treatment. However, recurrence occurs in about 70% of patients (3). Systemic therapy is the standard treatment for patients with inoperable or recurrent disease, such as sorafenib, lenvatinib, and immune checkpoint inhibitor (4). However, the prognosis of these patients are poor, with a 5-year survival rate of <8% (5). Thus, it is an urgent need to find new biomarkers for the diagnosis, treatment, and prognosis.

Cell division cycle-associated protein 2 (CDCA2) is a member of cell cycle-related proteins. It is reported that CDCA2 plays a role in the regulation of protein phosphatase 1(PP1) γ-dependent DNA damage response (DDR) by forming a complex with PP1γ (6). In addition, CDCA2 regulates H3 phosphorylation in a PP1 dependent manner (7). CDCA2 promotes the tumorigenesis and development of prostate cancer, malignant melanoma, renal cancer, and other malignant tumors by promoting the proliferation of tumor cells (6, 8–10). CDCA2 participates in cell cycle regulation. It was reported that CDCA2 expression level affected the activation of DNA damage checkpoint. Cell cycle checkpoints are induced by DNA damage and cause cell cycle arrest (11, 12). Thus, CDCA2 plays an important role in the regulation of cell cycle progression. Previous studies have shown that CDCA2 is upregulated and associated with poor prognosis in some tumors, such as lung cancer (13), breast cancer (14), and pancreatic cancer (15). However, there are few reports about the correlation between CDCA2 expression and the clinicopathological characteristics of HCC.

To explore the expression pattern and the prognostic value of CDCA2 in HCC, we performed the current study.

## Methods

### Datasets and Clinical Information

Cell division cycle-associated protein 2 expression data of normal liver tissue (50 cases) and HCC tissues (374 cases), and the clinical data of patients with HCC were downloaded from The Cancer Genome Atlas database (TCGA-LIHC). The expression information of CDCA2 and patient information used in the current study were obtained from public database and therefore ethical approval was not required. R software (version 3.6.3) was used to perform the analysis. The difference of expression is visualized by dot graphs and box graphs.

### RNA Extraction and Quantitative Real-Time PCR Analysis of CDCA2 Expression in HCC Cell Lines

Hepatocellular carcinoma cell lines HepG2 and SNU182 and normal liver cell line THLE-3 were purchased from American Type Culture Collection (ATCC) cell bank. Total RNA of the cell lines was extracted using the TRIzol reagent (Invitrogen, Carlsbad, CA, USA) and reverse transcription was performed to obtain cDNA. Primer sequences of CDCA2 were shown as follows: forward, 5′-ATGACCGGCTGTCTGGAAT-3′, and reverse, 5′-GCTGAGACCTTCCTTTCTGGT-3′. According to the instructions of manufacturer of the SYBR Green reagent (ABI, CA, USA), quantitative real-time PCR (qRT-PCR) was performed to examine the expression of CDCA2 mRNA.

### Verification of CDCA2 Expression and Its Correlation With Survival by GEO Datasets and Kaplan–Meier Plotter

Microarray data and RNA sequencing data were downloaded from GEO database. The terms, such as “liver,” or “hepatocellular” and “cancer,” “carcinoma,” or “neoplasm” were used for the search. GSE27150, GSE54236, GSE56140, GSE64041, and GSE76427 were downloaded. GSE56140, GSE76427, and GSE64041 were used to validate the CDCA2 expression difference between normal tissues and HCC tissues. GSE27150, GSE54236, and GSE76427 were used to validate the relationship between CDCA2 expression and survival. Meta-analysis was performed to verify the hazard ratio (*HR*) of CDCA2 expression to survival. The combined value was calculated by *HR* and 95% *CI*. Heterogeneity between datasets was assessed by using the τ^2^ and *I*^2^ test. If *I*^2^ > 50%, the random-effects model was used, otherwise, the fixed-effects model was used. HCC data from Kaplan-Meier plotter database (https://kmplot.com/analysis/) were used to validate the relationship between CDCA2 expression and survival.

### Gene Set Enrichment Analysis

Patients were classified as CDCA2-high group and CDCA2-low group, using the median expression level of CDCA2 as cutoff value. Gene set enrichment analysis (GSEA) was conducted to assess the potential mechanism of CDCA2 in HCC. The c2.cp.kegg.v6.2.symbols.gmt was used as reference gene set. Parameter of gene set permutation for each analysis was 1,000. The significance of enriched gene sets was estimated by nominal *p*-value and false discovery rate (FDR) Q-value.

### Statistical Analysis

Statistical analyses were conducted by using R software (version 3.6.3). Results were considered as statistically significant if *p* < 0.05. First, the expression of CDCA2 in normal tissues and tumor tissues was compared by Wilcoxon rank sum test. The correlation between CDCA2 expression and clinicopathological characteristics was examined by logistic regression analysis. Then, the relationship between CDCA2 expression and survival in HCC was estimated by Kaplan–Meier method. The prognostic value of CDCA2 in HCC was estimated by the univariate and multivariate Cox regression analysis.

## Results

### CDCA2 Was Overexpressed in HCC Tissues

**Figure 1:**
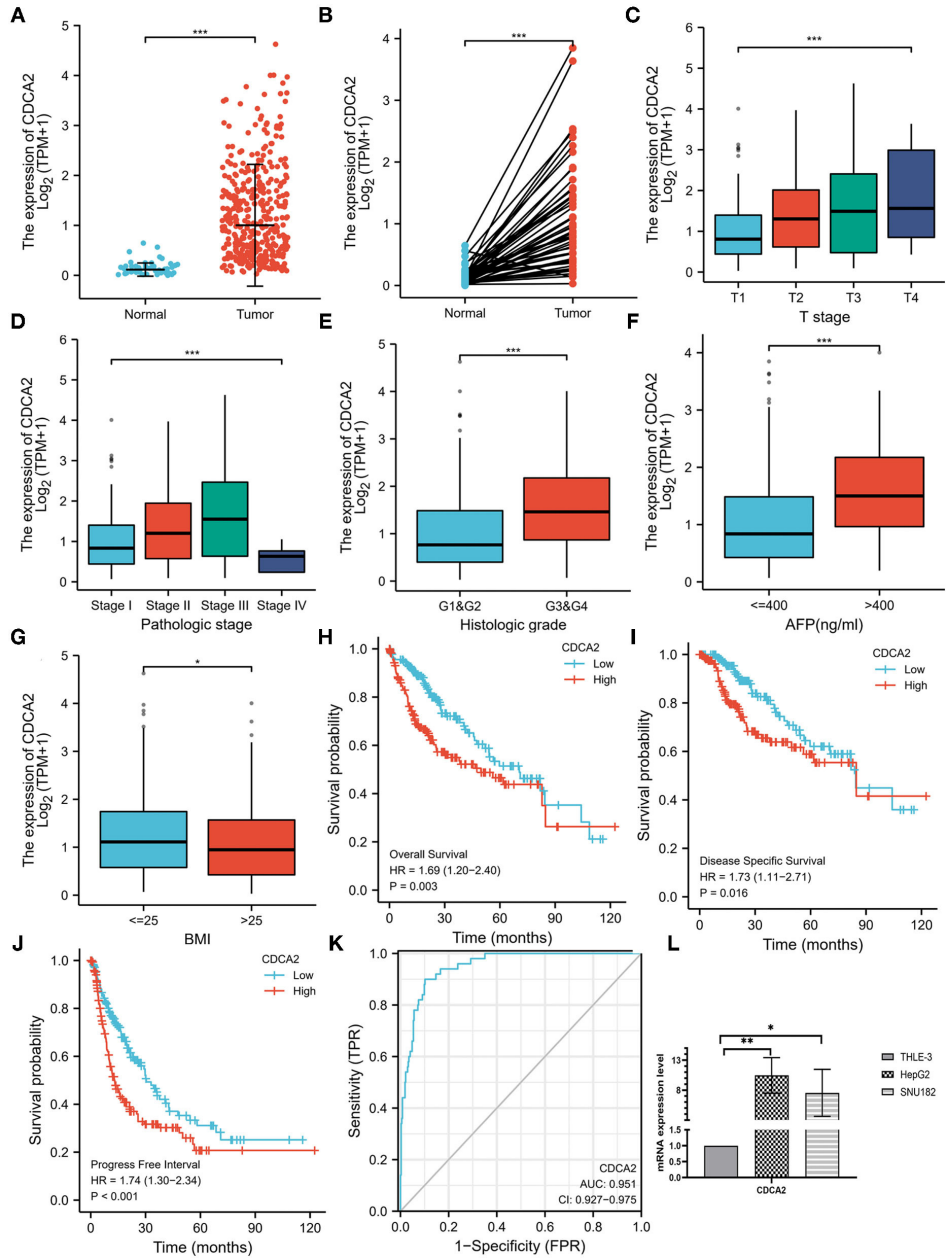
Cell division cycle-associated protein 2 (CDCA2) expression in hepatocellular carcinoma (HCC) and the correlations between CDCA2 expression and clinicopathological characteristic. **(A)** Cell division cycle-associated protein 2 expression in HCC tissues and normal tissues (Wilcoxon rank sum test, ***p < 0.001). **(B)** Cell division cycle-associated protein 2 expression in HCC tissues and adjacent noncancerous tissues (Wilcoxon signed rank test, ***p < 0.001). **(C)** Expression level of CDCA2 in patients with different T stages (Kruskal–Wallis test, ***p < 0.001). **(D)** Expression level of CDCA2 in patients with different pathological stages (Kruskal–Wallis test, ***p < 0.001). **(E)** Expression level of CDCA2 in patients with different histologic grades (Wilcoxon rank sum test, ***p < 0.001). **(F)** Expression level of CDCA2 in patients with different AFP levels (Wilcoxon rank sum test, ***p < 0.001). **(G)** Expression level of CDCA2 in patients with different body mass index (BMI) (Wilcoxon rank sum test, ***p < 0.05). **(H)** Overall survivals of patients with high and low CDCA2 expression (log-rank test, *p* = 0.003). **(I)** Disease specific survivals of patients with high and low CDCA2 expression (log-rank test, *p* = 0.016). **(J)** Progress free intervals of patients with high and low CDCA2 expression (log-rank test, *p* < 0.001). **(K)** A receiver operating characteristic (ROC) curve and the area under the curve (AUC) of CDCA2 in HCC. **(L)** Cell division cycle-associated protein 2 mRNA was upregulated in both HepG2 and SNU182 cell lines (*t*-test, *p < 0.05, **p < 0.01).

The expression level of CDCA2 in 50 adjacent noncancer tissues and 374 HCC tissues was compared. It was shown that expression of CDCA2 was significantly higher in HCC tissues (*p* < 0.001) (Figure 1A). In fifty pairs of adjacent noncancerous and HCC tissues, CDCA2 expression was increased in HCC tissues in comparison with noncancerous tissues (*p* < 0.001) (Figure 1B). In short, CDCA2 was overexpressed in HCC tissues.

### CDCA2 Was Upregulated in HCC Cell Lines

To verify the upregulation of CDCA2 expression in HCC, we compared the expression of CDCA2 mRNA in HCC cells lines (HepG2 and SNU182) and normal liver epithelial cell line (THLE-3). Results showed that CDCA2 mRNA was upregulated in both HepG2 and SUN182 cell lines (Figure 1L).

### Correlations Between CDCA2 Expression Level and Clinicopathological Characteristics in Patients With HCC

Expression of CDCA2 in patients with HCC with different clinicopathological characteristics was analyzed. As shown in Figures 1C,D, the expression level of CDCA2 was increased as T stage (Kruskal–Wallis test, *p* < 0.001) and pathologic stage (Kruskal–Wallis test, *p* < 0.001) was increased. CDCA2 expression level in poorly differentiated groups (G3 and G4) was significantly higher than that in well differentiated groups (G1 and G2) (*p* < 0.001) (Figure 1E). Patients with high AFP level (*p* < 0.001) (Figure 1F) and low body mass index (BMI) (*p* < 0.05) (Figure 1G) also had higher CDCA2 expression level. Logistic regression analysis was performed to estimate the relationships between CDCA2 expression and clinicopathological characteristics of patients with HCC. It was revealed that an increased CDCA2 expression was significantly related to age [for >60 years vs. < =60 years, odds ratio (*OR*) = 0.505; 95% *CI*, 0.333–0.761, *p* = 0.001], T stage (for T2–T4 vs. T1, *OR* = 2.541; 95% *CI*, 1.678–3.875, *p* < 0.001), pathological stage (for stage II-IV vs. stage I, OR=2.359; 95%CI, 1.541–3.636, *p* < 0.001), AFP level (for >400 ng/ml vs. <400 ng/ml, *OR* = 3.558; 95% *CI*, 1.969–6.667, *p* < 0.001) and histologic grade (for G3–4 vs. G1–2, *OR* = 3.375; 95% *CI*, 2.170–5.314, *p* < 0.001) (Table 1).

**Table 1:** Logistic regression analysis was performed to estimate the relationships between cell division cycle-associated protein 2 (CDCA2) expression and clinicopathological characteristics.

**Characteristics**	**Total (*N*)**	**Odds Ratio (OR)**	***P*-value**
Age (>60 vs. ≤ 60)	373	0.505 (0.333–0.761)	**0.001** ^†^
Gender (Male vs. Female)	374	0.843 (0.545–1.300)	0.439
T stage (T2–T4 vs. T1)	371	2.541 (1.678–3.875)	**<0.001** ^†^
Pathologic stage (Stage II-IV vs. Stage I)	350	2.359 (1.541–3.636)	**<0.001** ^†^
BMI (>25 vs. ≤ 25)	337	0.817 (0.532–1.253)	0.355
AFP(ng/ml) (>400 vs. ≤ 400)	280	3.558 (1.969–6.667)	**<0.001** ^†^
Histologic grade (G3–4 vs. G1–2)	369	3.375 (2.170–5.314)	**<0.001** ^†^

† *p < 0.05*.

### Comparison of Survival in CDCA2-High and CDCA2-Low Patients

The median expression level of CDCA2 was 1.008 and it was used as the cutoff value. Patients with CDCA2 expression level higher than the cutoff value were considered as CDCA2 high expression, otherwise they were considered as CDCA2 low expression. Kaplan–Meier method was used to compare the survival of patients with high and low expression of CDCA2 from TCGA database. Patients with high CDCA2 expression level had worse overall survival (*HR* = 1.69; 95% *CI*, 1.20–1.40, *p* = 0.003), disease specific survival (*HR* = 1.73; 95% *CI*, 1.11–2.71, *p* = 0.016), and progress free interval (*HR* = 1.74; 95% *CI*, 1.30–2.34, *p* < 0.001) (Figures 1H–J).

### Verification of CDCA2 Overexpression in HCC by GEO Datasets

**Figure 2:**
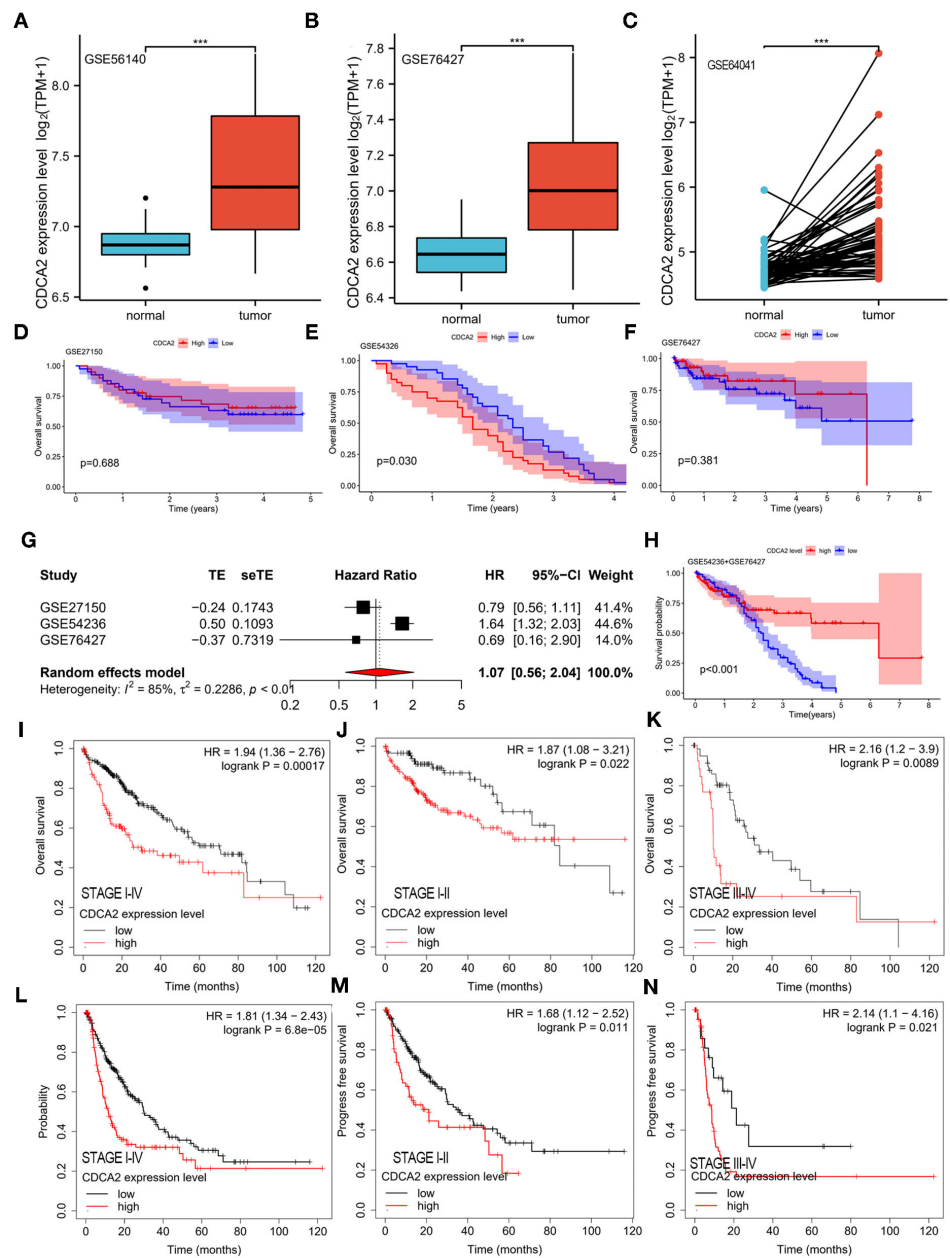
Verification of CDCA2 overexpression in HCC and the correlation between CDCA2 expression with survival by independent datasets. Cell division cycle-associated protein 2 expression in **(A)** GSE56140, **(B)** GSE76427, and **(C)** GSE64041. ***p < 0.001. The overall survivals of patients with high and low CDCA2 expression in **(D)** GSE27150, **(E)** GSE54326, and **(F)** GSE76427. **(G)** Meta-analysis of hazel ratio (*HR*) of CDCA2 overexpression to survival. **(H)** Overall survival of patients with high and low CDCA2 expression in GSE54236+GSE 76427 cohort. **(I)** Overall survivals of patients with high and low CDCA2 expression in Kaplan–Meier plotter database. **(J)** Overall survivals of stage I-II subgroup patients with high and low CDCA2 expression in Kaplan–Meier plotter database. **(K)** Overall survivals of stage III-IV subgroup patients with high and low CDCA2 expression in Kaplan–Meier plotter database. **(L)** Progress free survivals of patients with high and low CDCA2 expression in Kaplan–Meier plotter database. **(M)** Progress free survivals of stage I-II subgroup patients with high and low CDCA2 expression in Kaplan–Meier plotter database. **(N)** Progress freesurvivals of stage III-IV subgroup patients with high and low CDCA2 expression in Kaplan–Meier plotter database.

Cell division cycle-associated protein 2 expression in GSE56140, GSE76427, and GSE64041 was analyzed. The expression difference of CDCA2 between normal tissues and HCC tissues was compared. We found that CDCA2 expression was increased in HCC tissues (Figures 2A–C), which was consistent with the results of TCGA database.

### Verification the Relationship Between CDCA2 Expression and Survival in HCC

The survivals of patients with high and low CDCA2 expression in GSE27150, GSE54236, and GSE76427 were compared. In GSE54236 cohort, patients with high CDCA2 expression showed significantly worse survival than patients with low CDCA2 expression (*p* = 0.030) (Figure 2E). In GSE27150 and GSE76427 cohorts, survivals were not significantly different between CDCA2 high and CDCA2 low expression patients (Figures 2D,F). To further confirm the correlation of CDCA2 expression with survival in patients from GEO datasets, meta-analysis was conducted. Meta-analysis result of the GSE27150, GSE54236, and GSE76427 showed that CDCA2 overexpression was not associated with poor survival [combined *HR* = 1.07 (95% *CI*: 0.56–2.04)] (Figure 2G). After analyzing the result, we found that the homogeneity between studies was poor, with *I*^2^ = 85%. We further analyzed the array data of the three GEO datasets and found that GSE27150 did not provide normalization information and normalization method about the data. The poor homogeneity was mainly due to GSE27150. We excluded GSE27150 and performed survival analysis using the GSE54236 and GSE76427 datasets. The result indicated that high expression of CDCA2 was associated with better survival (*p* < 0.001) (Figure 2H). The result was inconsistent with the previous results from TCGA. We analyzed the data of the two datasets and we found that 69.3% of patients in high CDCA2 group were lost to follow-up while only 11.4% of patients in low CDCA2 group were lost to follow-up. The unbalanced loss of follow up rate between the two groups may affect the survival rate, and the high loss of follow-up rate in the high CDCA2 group may make the calculated survival rate higher than the actual survival rate. In addition, meta-analysis of the GSE54236 and GSE76427 showed that homogeneity between the two datasets was good (*I*^2^ = 27%) and increased CDCA2 was associated with poor clinical outcome (combined *HR* = 1.61 (95% *CI*:1.30–1.99). (Supplementary Figure S2). HCC data from Kaplan–Meier plotter database (https://kmplot.com/analysis/) were used to validate the relationship between CDCA2 expression and survival. It was indicated that patients with high CDCA2 expression showed poor overall survival (*HR* = 1.94; 95% *CI*, 1.36–2.76, *p* < 0.001) (Figure 2I) and progress free survival (*HR* = 1.81; 95% *CI*, 1.34–2.43, *p* < 0.001) (Figure 2L). In stage I-II subgroup and stage III-IV subgroup patients, CDCA2 overexpression was related to poor overall survival (for stage I-II subgroup, *HR* = 1.87; 95% *CI*, 1.08–3.21, *p* = 0.022; for stage III-IV subgroup, *HR* = 2.16; 95% *CI*, 1.20–3.90, *p* = 0.0089) (Figures 2J,K) and progress free survival (for stage I-II subgroup, *HR* = 1.68; 95% *CI*, 1.12–2.52, *p* = 0.011; for stage III-IV subgroup, *HR* = 2.14; 95% *CI*, 1.10–4.16, *p* = 0.021) (Figures 2M,N). The results were consistent with the results of TCGA database.

### Diagnostic and Prognostic Values of CDCA2 in HCC

A receiver operating characteristic (ROC) curve was plotted and the area under the curve (AUC) was calculated to examine the diagnostic value of CDCA2 in HCC. The ROC showed a sensitivity of 0.900 and a specificity of 0.898 and the AUC was 0.951 (Figure 1K). Univariate and multivariate analysis were used to estimate the correlation between clinicopathological characteristics and prognosis of HCC. Univariate analysis showed that CDCA2 expression (*HR* = 1.694; 95% *CI*, 1.196–2.401; *p* = 0.003), T stage (*HR* = 2.126; 95% *CI*, 1.481–3.052; *p* < 0.001), and pathologic stage (*HR* = 2.090; 95% *CI*, 1.429–3.055; *p* < 0.001) were related to poor survival (Table 2). Multivariate analysis showed that CDCA2 expression (*HR* = 1.613; 95% *CI*, 1.108–2.349; *p* = 0.013) was independently related to survival (Table 2). In summary, CDCA2 expression was an independent prognostic factor for HCC and increased CDCA2 expression was related to poor survival.

**Table 2:** Univariate and multivariate analysis were used to estimate the correlation between clinicopathological characteristics and prognosis of hepatocellular carcinoma (HCC).

		**Univariate analysis**		**Multivariate analysis**	
**Characteristics**	**Total (*N*)**	**HR** **(95% CI)**	***P*-value**	**HR** **(95% CI)**	***P*-value**
Age (>60 vs. < =60)	373	1.205 (0.850–1.708)	0.295		
Gender (Male vs. Female)	373	0.793 (0.557–1.130)	0.200		
BMI (>25 vs. < =25)	336	0.798 (0.550–1.158)	0.235		
T stage (T2–T4 vs. T1)	370	2.126 (1.481–3.052)	**<0.001** ^†^	0.731 (0.101–5.302)	0.757
Pathologic stage (Stage II–IV vs. Stage I)	349	2.090 (1.429–3.055)	**<0.001** ^†^	2.658 (0.362–19.545)	0.337
AFP(ng/ml) (>400 vs. < =400)	279	1.075 (0.658–1.759)	0.772		
Histologic grade (G4–3 vs. G1–2)	368	1.091 (0.761–1.564)	0.636		
CDCA2 (High vs. Low)	373	1.694 (1.196–2.401)	**0.003** ^†^	1.613 (1.108–2.349)	**0.013** ^†^

† *p < 0.05*.

### Overexpression of CDCA2 Was Related to Increased Expression of Immune Checkpoint

**Figure 3:**
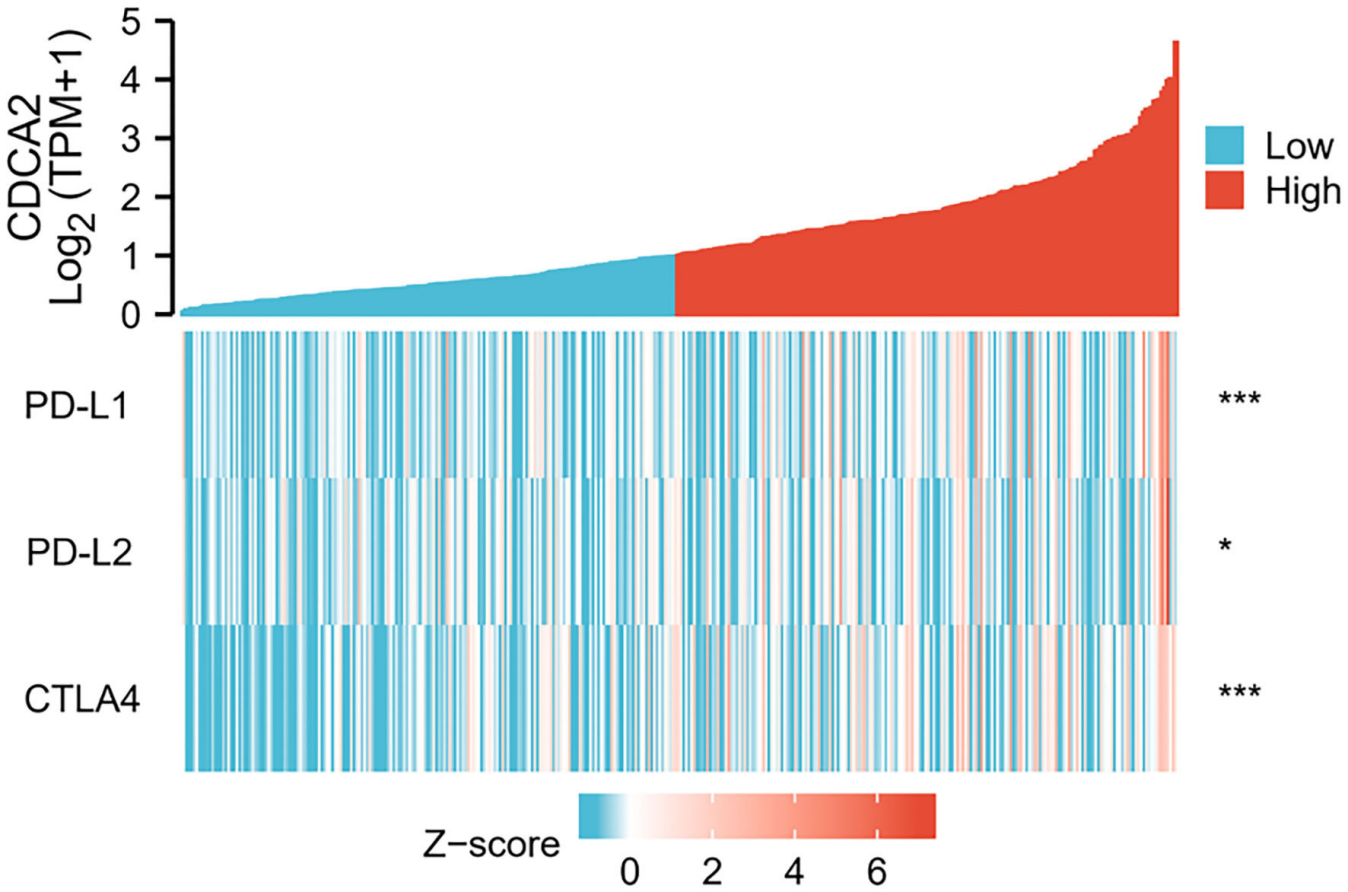
Correlation of CDCA2 with expression level of immune checkpoints. (*p < 0.05; ***p < 0.001).

Spearman's correlation analysis was performed to estimate the relation of CDCA2 expression to immune checkpoint expression, such as PD-L1, PD-L2, and CTLA4. The results showed that overexpression of CDCA2 was associated with increased expression of PD-L1 (Spearman's coefficient = 0.207, *p* < 0.001), PD-L2 (Spearman's coefficient = 0.118, *p* < 0.05), and CTLA4 (Spearman's coefficient = 0.355, *p* < 0.001) (Figure 3).

### Identification of CDCA2-Related Pathways

**Figure 4:**
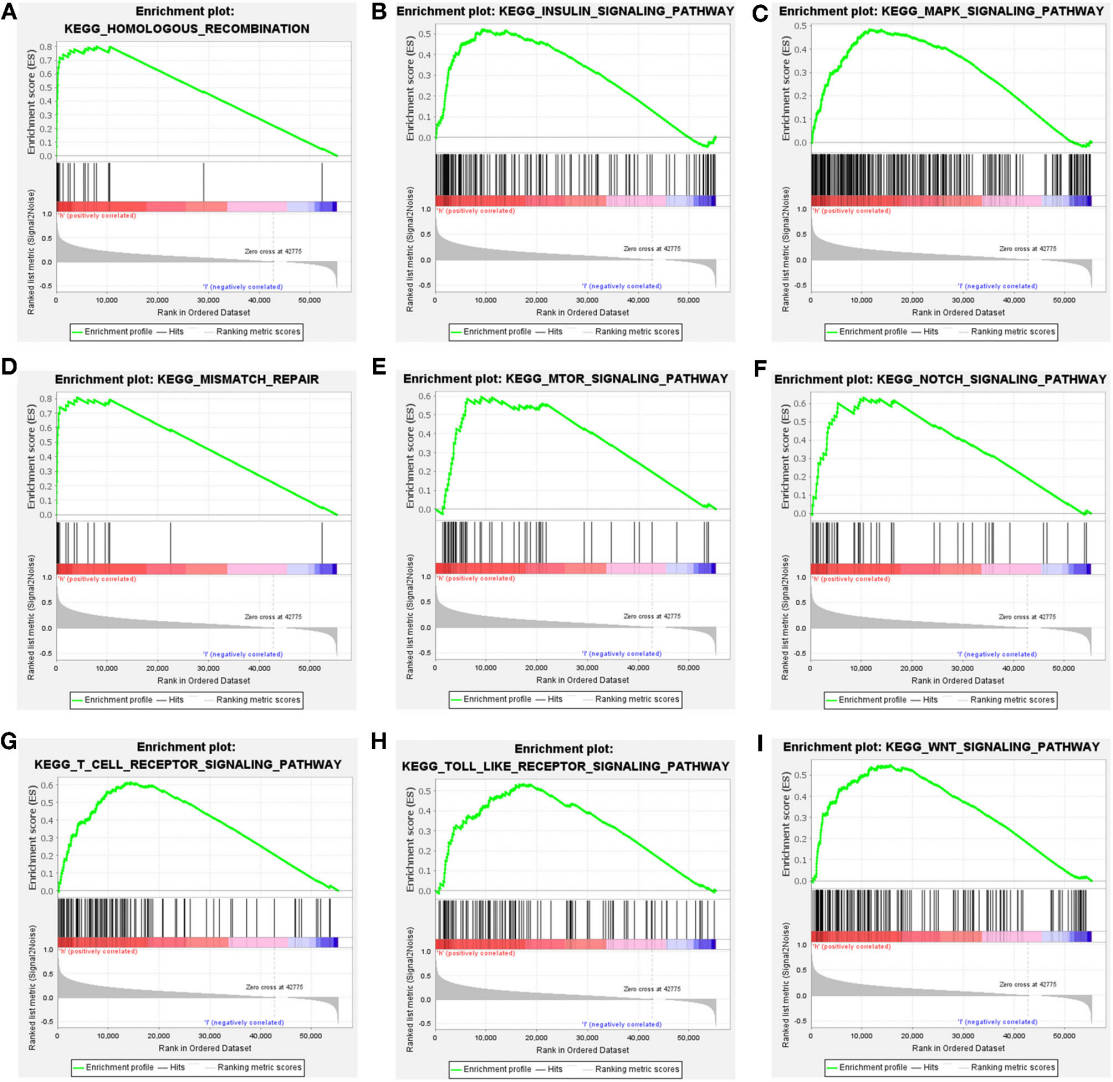
Cell division cycle-associated protein 2-related pathways identified by gene set enrichment analysis (GSEA). **(A)** Homologous recombination pathway, **(B)** insulin signaling pathway, **(C)** MAPK pathway, **(D)** mismatch repair pathway, **(E)** mTOR pathway, **(F)** Notch pathway, **(G)** T cell receptor pathway, **(H)** toll like receptor pathway, and **(I)** WNT pathway.

Patients were classified into CDCA2 high group and CDCA2 low group according to the median value of CDCA2 expression. CDCA2-related pathways were screened by GSEA. Results showed that homologous recombination pathway, insulin signaling pathway, mitogen-activated protein kinase (MAPK) pathway, mismatch repair pathway, mTOR pathway, Notch pathway, T cell receptor pathway, toll like receptor pathway, and WNT pathway were enriched in CDCA2 high expression phenotype (Table 3 and Figure 4).

**Table 3:** Cell division cycle-associated protein 2-related pathways screened by gene set enrichment analysis (GSEA).

**Gene set name**	**NES**	**NOM *p*-value**	**FDR *q*-value**
KEGG_HOMOLOGOUS_RECOMBINATION	1.99	0.000	0.003
KEGG_INSULIN_SIGNALING_PATHWAY	1.86	0.000	0.008
KEGG_MAPK_SIGNALING_PATHWAY	1.72	0.002	0.023
KEGG_MISMATCH_REPAIR	1.93	0.000	0.005
KEGG_MTOR_SIGNALING_PATHWAY	1.83	0.000	0.011
KEGG_NOTCH_SIGNALING_PATHWAY	1.85	0.004	0.010
KEGG_P53_SIGNALING_PATHWAY	2.10	0.000	0.000
KEGG_T_CELL_RECEPTOR_SIGNALING_PATHWAY	1.81	0.002	0.013
KEGG_TOLL_LIKE_RECEPTOR_SIGNALING_PATHWAY	1.74	0.002	0.021
KEGG_WNT_SIGNALING_PATHWAY	1.83	0.000	0.011

## Discussion

Cell division cycle-associated protein 2 participates in cell cycle regulation. It was reported that CDCA2 expression level affected the activation of DNA damage checkpoint. Cell cycle checkpoints are induced by DNA damage and cause cell cycle arrest (11, 12). CDCA2 participates in chromatin remodeling by regulating histone H3 de-phosphorylation (7). Thus, CDCA2 plays an important role in the regulation of cell cycle progression. Previous studies have shown that CDCA2 is upregulated and associated with poor prognosis in some tumors, such as lung cancer (13), breast cancer (14), and pancreatic cancer (15).

In the current study, we analyzed the expression pattern of CDCA2 and its diagnostic and prognostic value in HCC. To explore the potential mechanism by which CDCA2 regulates the tumorigenesis and development of HCC, we analyzed the CDCA2-high phenotype related signal pathways by GSEA. In the TCGA-LIHC cohort, we found that CDCA2 was upregulated in HCC and increased CDCA2 expression was associated with poor prognosis of patients with HCC. To validate the bioinformatic analysis results of the TCGA-LIHC cohort, we searched the GEO database and analyzed CDCA2 expression level in normal tissue and HCC tissue and its association with prognosis. We got consistent results with the results of TCGA-LIHC cohort.

Some reports have indicated that CDCA2 was associated with poor survival in HCC and the correlation between pathologic stage and histologic grade with CDCA2 expression was also reported (15–17). However, the relationship between CDCA2 expression and other clinical features was not analyzed. In the current study, we analyzed the correlation between CDCA2 expression level and clinicopathological features, such as T stage, lymph node invasion, distant metastasis, pathologic stage, histological grade, AFP level, and BMI. Logistic regression showed that CDCA2 expression was significantly associated with histological grade, AFP level, T stage, and pathologic stage. CDCA2 was increased as histological grade, AFP level, T stage, and pathologic stage increased. These results suggested that CDCA2 participated in the development of HCC. An ROC curve showed that CDCA2 had high diagnostic value for HCC, with an AUC of 0.951.

Univariate analysis showed that CDCA2 expression level, T stage, and pathologic stage may predict poor prognosis of HCC. Multivariate regression analysis further verified that CDCA2 had an independent prognostic value for HCC. The results were consistent with previous reports (15–17). Wang Y et al. demonstrated that low methylation of CDCA2 was related to poor survival. However, they did not study the relationship between CDCA2 expression level and clinical prognosis and the results were not verified by independent dataset (16). Though Wang Z also indicated that increased CDCA2 was related to poor survival in HCC, they did not verify the results by independent dataset (17). Wu B et al. showed that upregulation of CDCA2 was related to poor survival in HCC. They used only one dataset to validate the upregulation of CDCA2 and the correlation between CDCA2 expression and survival (15). However, the sample size was small. The validation dataset contained only 14 pairs of HCC tissues and adjacent tissues and 64 cases of patients with HCC (15). In the current study, we used three independent datasets to verify the upregulation of CDCA2 in HCC. The validation cohort contained larger sample size than previous study. GSE56140 contained 34 normal tissues and 35 tumor tissues. GSE76427 contained 52 normal tissues and 155 tumor tissues. GSE64041 contained 60 pairs of HCC tissues and adjacent tissues. The three independent datasets showed the consistent results. As these three datasets did not contain prognosis information, we used the other three datasets (GSE27150, GSE54236, and GSE76427) to validate the correlation between CDCA2 expression and clinical outcomes. The result from GSE27150 and GSE75427 showed that the prognosis of patients with high and low CDCA2 expression level did not have statistical difference. However, the result from GSE 54236 showed that patients with high CDCA2 expression had worse clinical outcome. The inconsistent results may be attributed to bias introduced by the small sample size in the GSE27150 and GSE76427 datasets. To further analyze the results, meta-analysis was performed. However, meta-analysis indicated that high CDCA2 expression was not related to poor survival. It should be noted that poor heterogeneity existed between the three datasets, with *I*^2^ = 85%. After the data of the three dataset, we found that GSE54236 and GSE76427 were both normalized by the same method (robust spline normalization, RSN) while normalization information of GSE27150 was not provided. After excluding GSE27150 and survival analysis of GSE54236 and GSE76427 by Kaplan–Meier method showed that increased CDCA2 was associated with better survival. The inconsistent result may be due to the obviously higher loss of follow-up rate in the high CDCA2 group. The meta-analysis result of the two datasets confirmed the high CDCA2 expression was related to poor survival.

Some research have studied the mechanisms of CDCA2 in HCC. It was reported that CDCA2 protected against oxidative stress by activating BRCA1-NRF2 pathways in HCC (18). Li et al. reported that CDCA2 promoted cell proliferation of HCC by activating AKT/CCND1 pathway (19). However, the relationship between immune checkpoint and CDCA2 expression has not been reported. As immune checkpoint inhibitors have become one of the standard treatments for HCC, we estimated whether the CDCA2 expression was related to immune checkpoint expression. Spearman's correlation analysis showed that increased expression of CDCA2 was associated with increased expression of immune checkpoints. It has been indicated that increased immune checkpoint was associated with inhibition of immune cells activity (20). The above results revealed that upregulation of CDCA2 may affect the prognosis by inhibiting immune cell activity.

Gene set enrichment analysis was performed to explore the potential mechanisms of CDAC2 in HCC. We found that homologous recombination pathway, insulin signaling pathway, MAPK pathway, mismatch repair pathway, mTOR pathway, Notch pathway, T cell receptor pathway, toll like receptor pathway, and WNT pathway were enriched in CDCA2 high expression phenotype. Homologous recombination pathway is a signal pathway associated with DNA double-strand breaks repair (21). Drugs targeting homologous recombination deficiency (HRD), such as poly(ADP-ribose) polymerase (PARP) inhibitors, have been proved to have an antitumor activity in some types of tumors, such as breast cancer and ovarian cancer (21, 22). Mismatch repair pathway is another DNA damage repair pathway, which promotes DNA damage response mediated by ataxia telangiectasia mutated (ATM) and ataxia-telangiectasia mutated (ATR) (23). The previous studies reported that BRCA1 was recruited by CDCA2 (18) and BRCA1 functioned in DNA repair process (24). These results supported that homologous recombination pathway and mismatch repair pathway were enriched in CDCA2 high phenotype. Insulin signaling pathway, which can be activated by IGF-1, promotes cell growth, proliferation, and inhibits apoptosis. It has been indicated that insulin signaling pathway activation was associated with increased risk of breast cancer (25) and colorectal cancer (26). MAPK pathway is the ubiquitous signal transduction pathway which involves in many processes of life and often alters in many disease (27). MAPK pathway regulates cellular activities during development of cancers, such as cell proliferation, cell apoptosis, and immune escape. Inhibiting the upstream kinase of MAPK pathway has become a therapeutic strategy of some cancer (28). The mTOR pathway involves in the regulation of protein synthesis, glucose metabolism, lipid metabolism, glutamine metabolism, and nucleotide synthesis in cancer cells. The mTOR pathway has become a therapeutic target for cancer therapy. mTOR inhibitors, such as rapamycin and everolimus, have been approved for the treatment of some types of cancers (29). It was reported that CDCA2 activated AKT related pathways and promoted HCC proliferation (19). mTOR was one of the downstream effectors of PI3K/AKT pathways (30). The current study showed that mTOR pathway was enriched in CDCA2 high phenotype. The result was consistent with previous reports. Notch pathway plays a vital role in promoting tumor development by changing tumor microenvironment and recruiting immunosuppressive cells (31). Moreover, Notch pathway can interact with WNT pathway and promote HCC development (32). Toll like receptors are important factors affecting the immune system and initiation of inflammatory response. It has been revealed that inhibiting toll like receptors suppresses the proliferation of HCC cells (33). The above finding results from GSEA provided information to explore the mechanism by which CDCA2 promoting the development of HCC.

However, some limitations existed in the current study. First, the number of tumor tissues in TCGA and GEO database was much higher than number of normal tissues, which were used as a control. Second, we only analyzed the CDCA2 mRNA expression of the tissue. The protein expression level of CDCA2 was not assessed. And finally, we only explored the potential involved pathways related to CDCA2 by bioinformatic analysis and the molecular mechanism was not explored in depth by molecular biology experiment. In addition, it should be noticed that the meta-analysis of the three GEO datasets indicated that CDCA2 was not associated with survival, though multiple Cox regression analyses pointed out that CDCA2 was associated with survival independently. The relationship between CDCA2 expression and survival should be validated clinically.

In conclusion, we analyzed the CDCA2 expression data of TCGA database and validated the results using independent cohorts from GEO database. The results showed that CDCA2 was increased in HCC and had a high diagnostic power for HCC. Kaplan–Meier analysis and univariate Cox regression analysis indicated that CDCA2 was associated with poor survival for HCC. Increased CDCA2 expression was associated with the upregulation of PD-L1, PD-L2, and CTLA4. In addition, we also screened the potential signal pathways related to CDCA2 in HCC. However, the prognostic value of CDCA2 in HCC needs further clinical exploration and validation.

## Public Database and Tools Used in the Current Study

1. The Cancer Genome Atlas (TCGA) database. Link: https://portal.gdc.cancer.gov/
2. Kaplan-Meier Plotter database. Link: https://kmplot.com/analysis/
3. Gene Expression Omnibus (GEO) database. Link: www.ncbi.nlm.nih.gov/geo/

## Data Availability Statement

The original contributions presented in the study are included in the article/Supplementary Material and access to datasets can be found here: The Cancer Genome Atlas (TCGA) database. Link: https://portal.gdc.cancer.gov/, Kaplan-Meier Plotter database. Link: https://kmplot.com/analysis/, Gene Expression Omnibus (GEO) database. Link: www.ncbi.nlm.nih.gov/geo/. Further inquiries can be directed to the corresponding author.

## Author Contributions

MT, ML, XA, and GH had full access to all the data in the study and take responsibility for the integrity of the data and the accuracy of the data analysis, designed the study, analysis and interpretation of data, and drafting and revising of the manuscript. MT and ML acquisition of data and statistical analysis. All authors contributed to the article and approved the submitted version.

## Conflict of Interest

The authors declare that the research was conducted in the absence of any commercial or financial relationships that could be construed as a potential conflict of interest.

## Publisher's Note

All claims expressed in this article are solely those of the authors and do not necessarily represent those of their affiliated organizations, or those of the publisher, the editors and the reviewers. Any product that may be evaluated in this article, or claim that may be made by its manufacturer, is not guaranteed or endorsed by the publisher.
