# Supplementary figures and images for: Increased CDCA2 Level Was Related to Poor Prognosis in Hepatocellular Carcinoma and Associated With Up-Regulation of Immune Checkpoints
Source: Front Med (Lausanne). 2022 Mar 7;8:773724. doi: 10.3389/fmed.2021.773724 (PMC8964461; doi:10.3389/fmed.2021.773724)

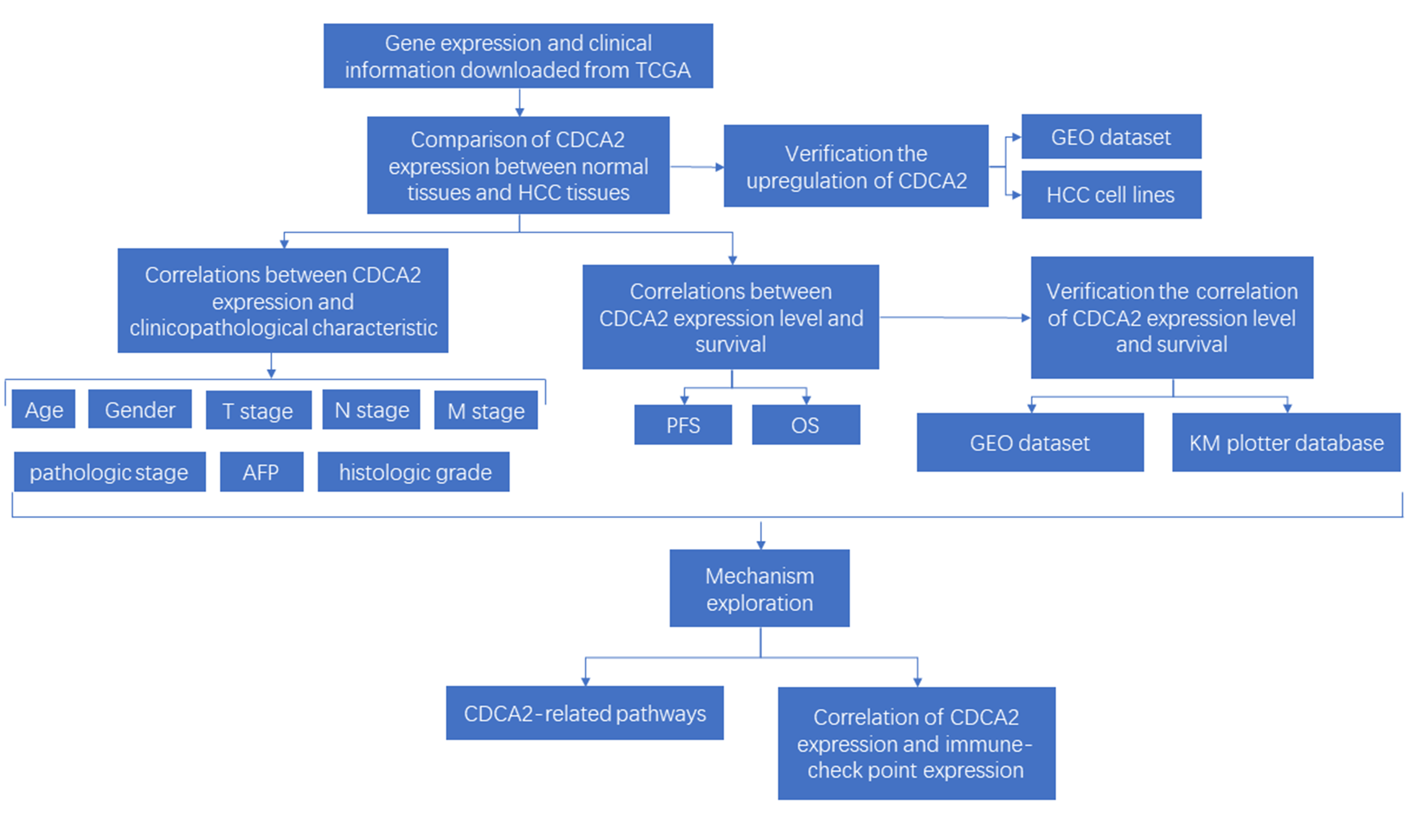

Supplement: Supplementary file 1 [file Image_1.TIF]

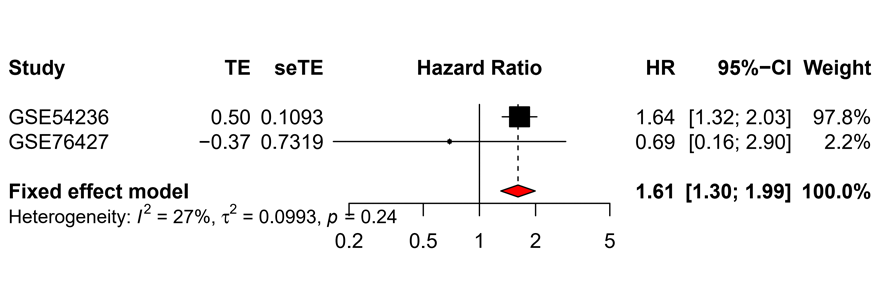

Supplement: Supplementary file 2 [file Image_2.TIF]
